# Akzidentelle Fixierung eines Pulmonalarterienkatheters während einer Herztransplantation

**DOI:** 10.1007/s00101-020-00852-0

**Published:** 2020-09-22

**Authors:** S. Bleiler, A. Holzamer

**Affiliations:** 1 Klinik für Anästhesiologie, Universitätsklinikum Regensburg, Franz-Josef-Strauss-Allee 11, 93053 Regensburg, Deutschland; 2 Klinik für Herz‑, Thorax- und herznahe Gefäßchirurgie, Universitätsklinikum Regensburg, Regensburg, Deutschland

**Keywords:** Herzchirurgie, Swan-Ganz-Katheter, Naht, Fluoroskopie, Resternotomie, Heart surgery, Swan-Ganz catheter, Suture, Fluoroscopy, Resternotomy

## Abstract

Die akzidentelle Fixierung eines Pulmonalarterienkatheters durch eine Naht ist eine seltene Komplikation. Dieser Fall handelt von einem Patienten zur Herztransplantation, bei dem der Katheter mittels Naht an der oberen venösen Kanülierungsstelle der Herz-Lungen-Maschine fixiert wurde. Nach Diagnostik mittels Fluoroskopie erfolgten die Resternotomie und die Lösung der Naht, woraufhin sich der Katheter entfernen ließ. Der Patient trug keine Folgeschäden davon.

## Abstract (en)

Inadvertent fixation of a Swan-Ganz pulmonary artery catheter by a suture is a rare complication. We present a case report of a patient undergoing heart transplantation in whom the catheter was fixed by a suture at the upper venous cannulation site for the cardiopulmonary bypass. After diagnostics with fluoroscopy the chest was reopened and the suture was released. The catheter could then be removed. The patient did not suffer from any consequential damage.

## Einleitung

Im Rahmen kardiochirurgischer Eingriffe kann laut der S3-Leitlinie „Intensivmedizinische Versorgung herzchirurgischer Patienten“ der Pulmonalarterienkatheter (PAK) bei Hochrisikopatienten eingesetzt werden, um zwischen einer rechts- oder linksventrikulären Dysfunktion unterscheiden zu können („Grade of Recommendation“ [GoR, Empfehlungsgrad] 0). Des Weiteren ist der PAK bei Patienten mit einem erhöhten Risiko für eine Rechtsherzdysfunktion indiziert (GoR B) [3].

Beim Einsatz des Swan-Ganz-Katheters ist eine Vielzahl von potenziellen Komplikationen beschrieben [3]. Diese sind in Auszügen in Tab. 1 dargestellt. Einen sehr seltenen Zwischenfall stellt die akzidentelle Fixierung des Katheters durch eine Naht dar, von dem wir hier berichten.

**Table 1:** 

Komplikation	Inzidenz (%)
Kardiale Arrhythmien	
*Supraventrikulär*	15
*Ventrikulär*	13–78
Schäden an Trikuspidal‑/Pulmonalklappe	0,5–2
Pulmonalarterienruptur	0,064–2
Lungeninfarkt durch nichtentleerten Ballon	0,8–1
Bakterielle Kontamination	11,6
Positive Blutkulturen	0,6
Septische Endokarditis	Unter 1,5
Nichtquantifizierbar: intravasale Knotenbildung, Fehllagen außerhalb der Westzone III, chirurgische Fixierung	

## Anamnese

Bei einem 37-jährigen, männlichen Patienten wurde am Universitätsklinikum Regensburg eine Herztransplantation aufgrund einer dilatativen Kardiomyopathie als Folge einer stattgehabten Myokarditis durchgeführt. Der Patient war im Jahr vor dem Eingriff mehrfach kardial dekompensiert und wies einen zunehmend schlechten Allgemeinzustand mit deutlicher kardialer Kachexie auf. Bei einer Körpergröße von 1,60 m betrug das Körpergewicht 50 kg (BMI 19,5 kg/m^2^). Der Patient zeigte sich allseits orientiert, ohne zentral- oder peripherneurologisches Defizit.

Der Patient war vor der Transplantation eine Woche im Status „high urgency (HU)“ gelistet.

In der Vorgeschichte ließ sich eine transitorische ischämische Attacke am ehesten kardioembolischer Natur erheben, weshalb der Patient mit Edoxaban antikoaguliert wurde. Des Weiteren wurde dem Patienten primärprophylaktisch 37 Monate vor der Herztransplantation ein „implantable cardioverter defibrillator“ (ICD) implantiert.

Im Rahmen der präoperativen Evaluation zeigte sich bei dem Patienten eine hochgradig reduzierte Pumpfunktion des dilatierten linken Herzens mit einer Ejektionsfraktion von ca. 15 %. Der rechte Ventrikel zeigte sich dilatiert mit reduzierter Kontraktilität (TAPSE 11 mm). Die präoperative Rechtsherzkatheteruntersuchung ergab einen Herzindex von 1,9 l/min und m^2^ KOF.

Unmittelbar vor der Herztransplantation wurden dem Patienten auf der herzchirurgischen „intermediate care unit“ kontinuierlich 6,7 μg/kgKG und min Dobutamin sowie 1,3 μg/kgKG und min Enoximon zur inotropen Unterstützung verabreicht.

Aufgrund der kardial bedingten Leberstauung fielen bereits erhöhte Leberfunktionsparameter auf (GPT 121 U/l, γ‑GT 316 U/l, alkalische Phosphatase 170 U/l). Die Nierenfunktion zeigte sich bei einem Kreatininwert von 1,93 mg/dl und einer glomerulären Filtrationsrate von 43 ml/min und 1,73 m^2^ KOF ebenfalls bereits eingeschränkt.

Der Patient war präoperativ bereits mit einem 4‑lumigen zentralen Venenkatheter (ZVK) in der rechten V. jugularis interna sowie einem Shaldon-Katheter in der rechten V. subclavia ausgestattet.

## Befund

Nach problemloser Anlage einer arteriellen Blutdruckmessung sowie Narkoseeinleitung mittels 0,1 mg/kgKG Midazolam, 1 μg/kgKG Sufentanil und 1 mg/h Rocuronium und anschließender Intubation erfolgte die problemlose Anlage einer 8,5-F-Schleuse mit 10 cm Länge (Fa. Arrow®, Teleflex®, Reading, PA, USA) in der linken V. jugularis interna in Seldinger-Technik. Zur Stabilisierung der Hämodynamik wurden zusätzlich zu der präoperativ etablierten inotropen Medikation kontinuierlich 0,17 μg/kgKG und min Noradrenalin verabreicht.

Weiterhin wurde ebenfalls in der linken V. jugularis interna auf 14 cm Hautniveau (alle weiteren Tiefenangaben beziehen sich ebenfalls auf das Hautniveau) ein 5‑Lumen-ZVK etabliert, um die postoperative Entfernung des ZVK auf der rechten Seite zu ermöglichen und die spätere Entnahme von Myokardbiopsien so zu erleichtern. Beide Katheterisierungen erfolgten ohne Komplikationen.

Anschließend wurde ein Swan-Ganz-Katheter (Fa. Edwards Lifescience, Irvine, CA, USA) mithilfe einer TwistLock™-Cath Gard®-Schutzhülle (Fa. Arrow®) auf 13 cm Tiefe eingeführt und dort in der Schleuse arretiert. Dies wurde als sicherer Abstand zu der Kanülierungsstelle in der V. cava superior zur Vermeidung einer Fixierung eingeschätzt.

Die bereits liegenden Katheter auf der rechten Körperseite wurden jeweils um ca. 5 cm zurückgezogen.

Die Herztransplantation verlief nach problemloser aortaler und bikavaler Kanülierung zur Etablierung eines extrakorporalen Kreislaufs unauffällig.

Nach Abschluss der Anastomosierung der Vorhöfe und der großen Gefäße wurde nach Entwöhnung von der Herz-Lungen-Maschine anästhesieseitig versucht, den PAK anhand der Druckkurve in die pulmonalarterielle Strombahn einzuschwemmen, was trotz mehrfacher Versuche bei problemlosem Kathetervorschub misslang. Es konnte keine rechtsventrikuläre oder pulmonalarterielle Druckkurve dargestellt werden. Beim Versuch, den Katheter weiter als 18 cm zurückzuziehen, zeigte sich ein federnder Widerstand.

Durch die transösophageale Echokardiographie (TEE) gelang aufgrund von Artefakten keine Darstellung des PAK.

## Diagnose

In Rücksprache mit dem Operateur wurden die möglichen Ursachen diskutiert. Dieser konnte in der V. cava superior eine Struktur tasten, die als Shaldon-Katheter interpretiert wurde. Die Möglichkeit einer akzidentellen Fixierung durch eine Naht wurde in Betracht gezogen, jedoch schienen andere Ursachen wahrscheinlicher (Knotenbildung, Obstruktion der Schleuse). Es wurde eine postoperative Bildgebung zur weiteren Evaluation vereinbart.

Um die kontinuierliche Überwachung der rechtsventrikulären Funktion zu gewährleisten, wurde problemlos eine weitere 8,5-F-Schleuse in der rechten V. jugularis interna angelegt, worüber die Einführung eines zweiten PAK in die pulmonalarterielle Strombahn gelang.

**Figure 1:**
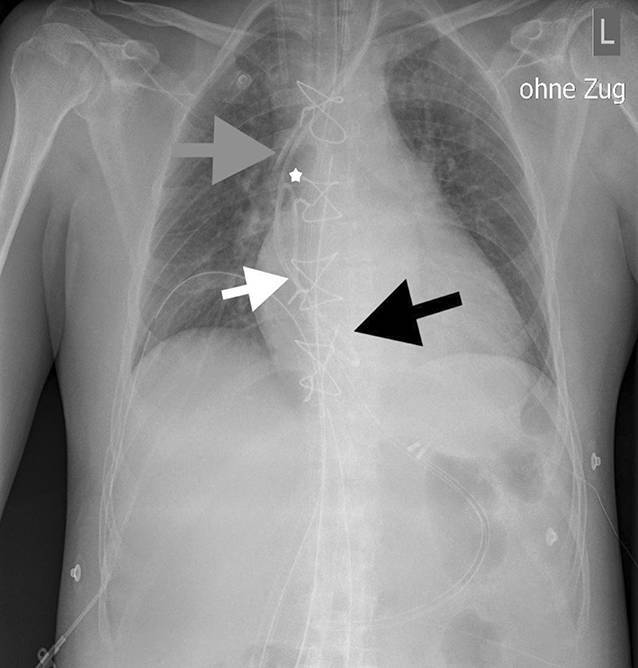

Am ersten postoperativen Tag wurden 2 Röntgenaufnahmen des Thorax erstellt, welche einerseits unter Kathetervorschub eine Schlingenbildung des von links eingeführten ersten PAK mit einer mutmaßlichen Umschlagsstelle in Höhe der V. cava superior zeigte (Abb. 1).

**Figure 2:**
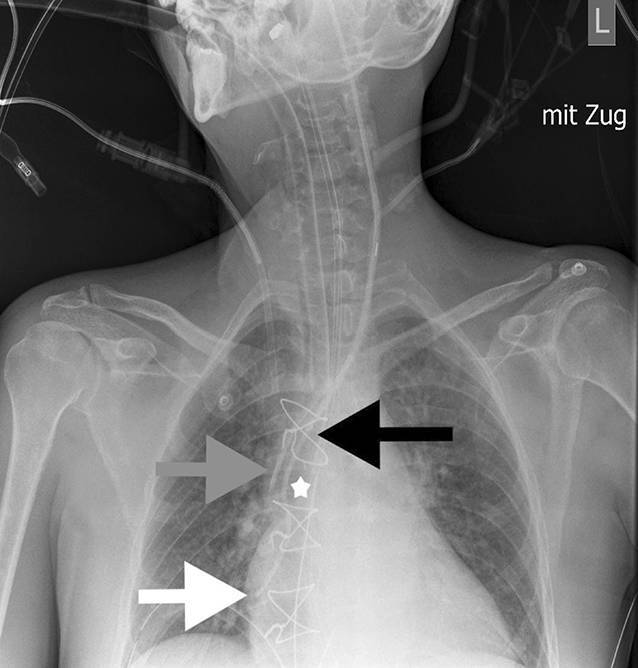

Andererseits stellte sich nach Rückzug des Katheters bis zum Auftreten des Widerstands ein normaler Katheterverlauf ohne Schlinge dar (Abb. 2).

Eine Knotenbildung konnte nicht beobachtet werden.

Der analgosedierte und beatmete Patient wurde zur Entfernung des linksseitig eingebrachten PAK erneut in den OP gebracht. Zunächst erfolgte die Sondierung mittels eines Führungsdrahtes unter Fluoroskopie (Durchleuchtung), während derer sich bei Kathetervorschub die Schlingenbildung durch eine Fixierung im Bereich der V. cava superior bestätigte und beim Rückzug ab 18 cm eine Zugübertragung auf das ganze Herz zeigte.

Dadurch erhärtete sich der Verdacht der Fixierung des Katheters im Bereich der oberen venösen Kanülierungsstelle.

## Therapie und Verlauf

Im Anschluss erfolgten daher die Resternotomie und das Lösen einer Naht an der Kanülierungsstelle, woraufhin sich der Katheter problemlos vollständig entfernen ließ.

**Figure 3:**
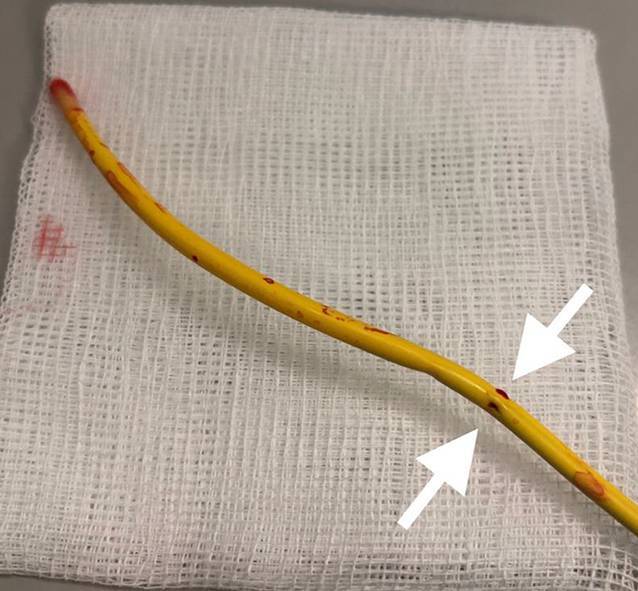

Nach der Entfernung ließen sich im distalen Abschnitt des Katheters deutlich 2 Perforationsstellen erkennen (Abb. 3).

Der Patient erlitt durch den 45-minütigen Zweiteingriff keinen relevanten Blutverlust. Der Katecholaminbedarf blieb mit 0,07 μg/kgKG und min Adrenalin und 0,04 μg/kgKG und min Noradrenalin über den gesamten Eingriff hinweg konstant auf dem präoperativem Niveau.

Der unmittelbare weitere Verlauf gestaltete sich bei guter Transplantatfunktion erfreulicherweise komplikationslos. So konnte der Patient zügig extubiert und am 5. postoperativen Tag auf die kardiochirurgische „Intermediate-care“-Station verlegt werden. Die Katecholamintherapie war rasch rückläufig und konnte zeitnah ausgeschlichen werden. Der neurologische Status zeigte sich unverändert zum präoperativen Befund. Nach 2 Wochen wurde der Patient in gutem Allgemeinzustand heimatnah zur stationären Weiterbehandlung verlegt.

## Diskussion

Der PAK hat trotz rückläufigem Einsatz und des Risikos potenzieller Komplikationen weiterhin einen hohen Stellenwert im erweiterten hämodynamischen Monitoring.

Die Herztransplantation zählt zu den Eingriffen, bei denen der Einsatz des PAK indiziert ist, da die isolierte Rechtsherzinsuffizienz die häufigste Ursache für ein frühes Transplantatversagen darstellt [7].

Der PAK ermöglicht über die Erfassung hämodynamischer Parameter des kleinen Kreislaufs (z. B. pulmonalarterieller Druck, pulmonalarterieller Widerstand) eine frühzeitige Diagnosestellung und zielgerichtete Therapie.

Des Weiteren ermöglicht er die Unterscheidung zwischen einer rechts- bzw. linksventrikulären Dysfunktion (z. B. über die Erfassung des pulmonalkapillären Verschlussdrucks als Parameter für die linksventrikuläre Vorlast bzw. die kontinuierliche Berechnung der rechtsventrikulären Ejektionsfraktion bei Verwendung von modernen Thermodilutionskathetern) [3, 7].

Die akzidentelle Fixierung eines Katheters im Rahmen herzchirurgischer Eingriffe ist eine sehr seltene Komplikation, wenngleich sich auch ähnliche Fälle in der Literatur finden lassen [2, 5]. Allerdings ist nach Meinung der Autoren von einem „underreporting bias“ auszugehen, d. h., dass die tatsächliche Inzidenz dieser Komplikation nicht vollkommen erfasst wird.

Falls es beim Einsatz eines PAK zu einem Widerstand während des Zurückziehens des Katheters kommt, ist eine umfangreiche Ursachenabklärung zur Vermeidung fataler Konsequenzen zu empfehlen. Größere Kraftanstrengungen zur Überwindung des Widerstands sollten dringend vermieden werden, da dies zu Gefäßrupturen mit deletärem Ausgang führen kann [4].

Diagnostisch können das Thoraxröntgen, die TEE und die Fluoroskopie (Durchleuchtung) eingesetzt werden [6]. Beide radiologischen Methoden kamen auch in unserem Fall zum Einsatz und bestätigten durch die Visualisierung der Schlingenbildung und des Zugs am Herzen die Differenzialdiagnose.

Wie bereits oben erwähnt, konnte mittels TEE aufgrund von Artefakten in diesem Fall keine Aussage über die Ursache getroffen werden.

Die postoperativen Röntgenbilder zeigen die Spitze des PAK deutlich tiefer als jene des 5‑Lumen-ZVK. Aufgrund des initialen Vorschubs der Katheter (13 cm vs. 14 cm; s. oben) wären allerdings beide Spitzen auf ungefähr gleicher Höhe zu erwarten gewesen.

Dies legt den Verdacht nahe, dass die Fixierung nicht durch die Kanülierungsnaht, sondern erst im Verlauf nach Entfernung der oberen venösen Kanüle erfolgte. Ursächlich war wahrscheinlich eine Übernähung der Kanülierungsstelle zur Blutstillung, die zeitgleich zum Kathetervorschub vorgenommen wurde.

Retrospektiv betrachtet, hätte eine intraoperative radiologische Bildgebung eine Resternotomie und die damit potenziell erhöhte Morbidität mit hoher Wahrscheinlichkeit vermeiden können [1].

Weiterhin zeigen die Röntgenbilder die mutmaßliche Anheftungsstelle des PAK im Bereich der Spitze des ebenfalls von links eingebrachten 5‑Lumen-ZVK, sodass der als sicher eingeschätzte Vorschub auf 14 cm Hautniveau bereits als zu tief zu betrachten ist. Vor allem bei geringer Körpergröße in Verbindung mit einer tiefen Punktion und hoher Kanülierungsstelle ist ein geringerer Vorschub zur Vermeidung einer Katheterfixierung nötig.

## Fazit für die Praxis

- Sollte ein Pulmonalarterienkatheter nicht ohne Widerstand zu bewegen sein, muss im Rahmen herzchirurgischer Eingriffe an eine akzidentelle Fixierung durch chirurgische Nähte gedacht werden.
- Zur Vermeidung einer Resternotomie muss die Klärung des Verdachts auf einen angenähten Katheter zwingend noch intraoperativ erfolgen.
- In der Herzchirurgie sollte vor den Phasen mit erhöhtem Risiko für eine akzidentelle Fixierung (Etablierung/Entwöhnung von der Herz-Lungen-Maschine (HLM), Übernähungen zur Blutstillung, Anastomosen) der Pulmonalarterienkatheter in eine sichere Distanz (a.e. in die Schleuse) zurückgezogen werden.
- Die interdisziplinäre Kommunikation kann hier maßgeblich zur Vermeidung der Fixierung von Kathetern beitragen.
